# Working memory capacity and emotion regulation strategies differentially predict proactive and reactive cognitive control

**DOI:** 10.3389/fpsyg.2026.1798190

**Published:** 2026-07-02

**Authors:** Ping Xu, Junming Qi, Wenchang Wen, Juan Zhang, Bo Hu, Yu Chen

**Affiliations:** 1 College of Health and Intelligent Engineering, Chengdu Medical College, Chengdu, China; 2 Sichuan Provincial Key Laboratory of Philosophy and Social Sciences for Intelligent Medical Care and Elderly Health Management, Chengdu Medical College, Chengdu, China

**Keywords:** cognitive reappraisal, inhibitory control, proactive control, stop-signal task, working memory capacity

## Abstract

**Introduction:**

Extensive evidence suggests that both working memory (WM) and emotion regulation shape cognitive control, yet their distinct contributions to proactive and reactive control remain unclear.

**Methods:**

Guided by the dual mechanisms of control (DMC) framework, sixty-six healthy participants completed a change detection task to assess working memory capacity (WMC), a modified stop-signal task (SST) to index proactive and reactive control, and the Emotion Regulation Questionnaire (ERQ) to evaluate habitual regulation strategies.

**Results:**

Correlation and regression analyses revealed differential predictive patterns. The proactive control (PC effect), quantified as the reaction time difference between Uncertain-Go and Certain-Go trials in the SST task, was significantly related to WMC, with a marginal contribution from expressive suppression. Reactive control, as indexed by stop-signal reaction time (SSRT), was significantly associated with cognitive reappraisal strategy. Expressive suppression strategy emerged as a significant predictor of SSRT only within the regression model, indicating a potential suppressor effect. Furthermore, a positive correlation between the PC effect and SSRT indicated partial interdependence between proactive and reactive control.

**Discussion:**

These results demonstrate that working memory and emotion regulation strategies exert distinct, yet complementary, influences on cognitive control, highlighting a dynamic interplay between cognitive and affective systems in goal-directed behavior.

## 1 Introduction

Cognitive control is a core component of adaptive behavior, enabling individuals to regulate thoughts, emotions, and actions in accordance with internal goals and changing environmental demands. One of the most influential theoretical models is the dual mechanisms of control (DMC) framework, which posits two complementary but interacting modes of control: proactive and reactive (Braver, 2012; Braver et al., 2021; Tang et al., 2022). Proactive control involves the sustained maintenance of goal-relevant representations to guide behavior in anticipation of upcoming task demands, whereas reactive control entails the transient, stimulus-driven reactivation of task goals in response to conflict or interference (Braver, 2012; Cole et al., 2012; Badre, 2025). Optimal cognitive regulation emerges from a dynamic interplay between these two modes, which may operate independently yet adaptively interact depending on contextual contingencies and individual cognitive profiles (Braver, 2012). An important yet unresolved question concerns the unique predictors of proactive versus reactive control. Evidence suggests that higher-order cognitive capacities, particularly working memory (WM) and emotion regulation strategies, may differentially influence these two control modes (Rosales et al., 2022; Manippa et al., 2023). Understanding how these factors predict individual differences in proactive and reactive control has important theoretical implications for the DMC framework.

WM, defined as the capacity to actively maintain and manipulate task-relevant information (Engle, 2002; Kane and Engle, 2002; Kane and Engle, 2003; Sohn and Doane, 2003; Rosales et al., 2022), is theoretically linked to proactive control through shared cognitive demands. Proactive control requires the sustained maintenance of goal representations during preparatory periods, which constitutes a core function of WM. Individuals with higher working memory capacity (WMC) are therefore expected to more effectively sustain task-relevant information, enabling more robust proactive engagement (Richmond et al., 2015; Tang et al., 2022; Zhang and Wang, 2022). In contrast, the relationship between WMC and reactive control remains less clear, as reactive control primarily involves transient, stimulus-driven activation rather than sustained maintenance processes. To precisely quantify individual differences in WMC, the Change Detection Task (CDT) was most used in recent research, which allows for the estimation of an individual’s WM storage capacity by requiring the maintenance of visual items (Daniel et al., 2016; Kotyusov et al., 2023; Zhao et al., 2023).

Emotion regulation strategies may also differentially influence cognitive control modes, though the mechanisms remain incompletely understood. Emotion regulation encompasses cognitive and behavioral strategies that modify the trajectory or experience of emotional states, including cognitive reappraisal and expressive suppression (Gross, 1998; Cutuli, 2014; Gross, 2015; Debra et al., 2024; Rónai et al., 2024). Cognitive reappraisal, an antecedent-focused strategy that involves reinterpreting emotional stimuli, has been associated with reduced emotional interference and enhanced cognitive control (Ochsner and Gross, 2005; Rincón-Pérez et al., 2025). By efficiently managing emotional responses early in the emotion generation process, cognitive reappraisal may preserve cognitive resources that can be allocated to cognitive control tasks. In contrast, expressive suppression, a response-focused strategy that involves inhibiting emotional expressions after they arise, imposes sustained cognitive costs (Butler et al., 2003; John and Gross, 2004; Schmeichel et al., 2008; Li et al., 2021a). The habitual use of expressive suppression may engage similar inhibitory processes as those required for cognitive control, potentially being associated with control mode preferences through overlapping neural mechanisms (Richards and Gross, 2000; Ochsner and Gross, 2005). However, whether emotion regulation strategies and WM differentially predict proactive and reactive control remains unclear.

Despite the theoretical and empirical importance of these factors, there is a paucity of research integrating both emotion regulation and WM to examine their joint and differential contributions to proactive and reactive control. Incorporating these constructs within the DMC framework offers a more comprehensive account of how affective regulation strategies and cognitive resource constraints jointly shape control mode efficiency. Addressing this gap, the present study employed a modified stop-signal task (SST) to investigate how emotion regulation strategies and WM differentially predict individual differences in proactive and reactive control (Rosales et al., 2022; Zhang and Wang, 2022; Kotyusov et al., 2023; Manippa et al., 2023).

The SST is a well-validated paradigm for quantifying inhibitory control, wherein participants are required to respond rapidly to Go stimuli but inhibit responses upon presentation of a stop signal (Logan and Cowan, 1984; Verbruggen and Logan, 2008b; Chikazoe et al., 2009; Manippa et al., 2023). Response inhibition is typically operationalized as the stop-signal reaction time (SSRT), which indexes reactive control efficiency. However, conventional SST designs provide limited indices of proactive control (Verbruggen and Logan, 2008a; Chikazoe et al., 2009). To address this limitation, we modified the classic task by introducing a baseline condition to differentiate between Certain-Go trials, which guaranteed no stop signal would appear, and Uncertain-Go trials, which carried a potential stop signal. The reaction time (RT) difference between these conditions constitutes the proactive control (PC effect), reflecting the degree to which participants strategically adjust response timing to maintain goal readiness under potential stopping demands (Gao et al., 2024; Ziri et al., 2024). As a well-established behavioral marker, the PC effect has been reliably utilized to investigate the neurobiological underpinnings of proactive control (Gao et al., 2024; Ziri et al., 2024). For instance, recent evidence involving children with ADHD has validated this measure by linking it to disrupted neural coding within the salience and fronto-parietal networks (Gao et al., 2024). This paradigm therefore enables concurrent assessment of both proactive control (via PC effect) and reactive control (via SSRT) within a unified experimental framework.

In summary, the present study aimed to elucidate how WM and emotion regulation strategies differentially predict proactive and reactive control. Grounded in the dual mechanisms of control framework (Braver, 2012; Friehs et al., 2021; Viviani et al., 2024) and emotion regulation theory (Ochsner and Gross, 2005; Gross, 2015; Manippa et al., 2023), we hypothesized that: (1) WM would predict proactive control efficiency, as indexed by the PC effect; (2) emotion regulation strategies would differentially predict cognitive control modes, with cognitive reappraisal predicting reactive control efficiency (SSRT) and expressive suppression showing associations with both proactive and reactive control; and (3) proactive and reactive control would show interdependence, as reflected in a positive relationship between the PC effect and SSRT.

## 2 Materials and methods

### 2.1 Participants

A total of 66 healthy adults (age range: 18–26 years; 33 females) were recruited for the present study. All participants reported normal or corrected-to-normal visual acuity, intact color vision, and no history of neurological, psychiatric, or cardiovascular disorders. Prior to participation, each individual provided written informed consent and completed an adult safety screening questionnaire. Participants received monetary compensation upon completion of the experiment. The study was approved by the Biomedical Ethics Committee of Chengdu Medical College and was conducted in strict accordance with the Declaration of Helsinki.

### 2.2 Experiment task

#### 2.2.1 Emotion regulation strategies

Participants’ habitual emotion regulation tendencies were assessed using the revised Emotion Regulation Questionnaire (ERQ) (Gross and John, 2003; Gouveia et al., 2018; Manippa et al., 2023). The ERQ consists of 10 items rated on a 7-point Likert scale (1 = strongly disagree, 7 = strongly agree) and is comprised of two independent subscales. The cognitive reappraisal subscale (6 items: 1, 3, 5, 7, 8, 10) evaluates the propensity to regulate emotions by reinterpreting the meaning of situations (e.g., “When I want to feel less negative emotion, I change the way I’m thinking about the situation”), while the expressive suppression subscale (4 items: 2, 4, 6, 9) measures the tendency to inhibit outward emotional expression (e.g., “I control my emotions by not expressing them”). The score for each subscale was calculated as the mean of its respective items, with higher scores indicating a greater frequency of using that strategy. The Chinese version of the ERQ has demonstrated good reliability and validity in Chinese populations (Li et al., 2021a,b). In the present study, internal consistency was satisfactory for both subscales (Cronbach’s *α* = 0.784 for cognitive reappraisal and *α* = 0.728 for expressive suppression).

#### 2.2.2 Change detection task

**Figure 1:**
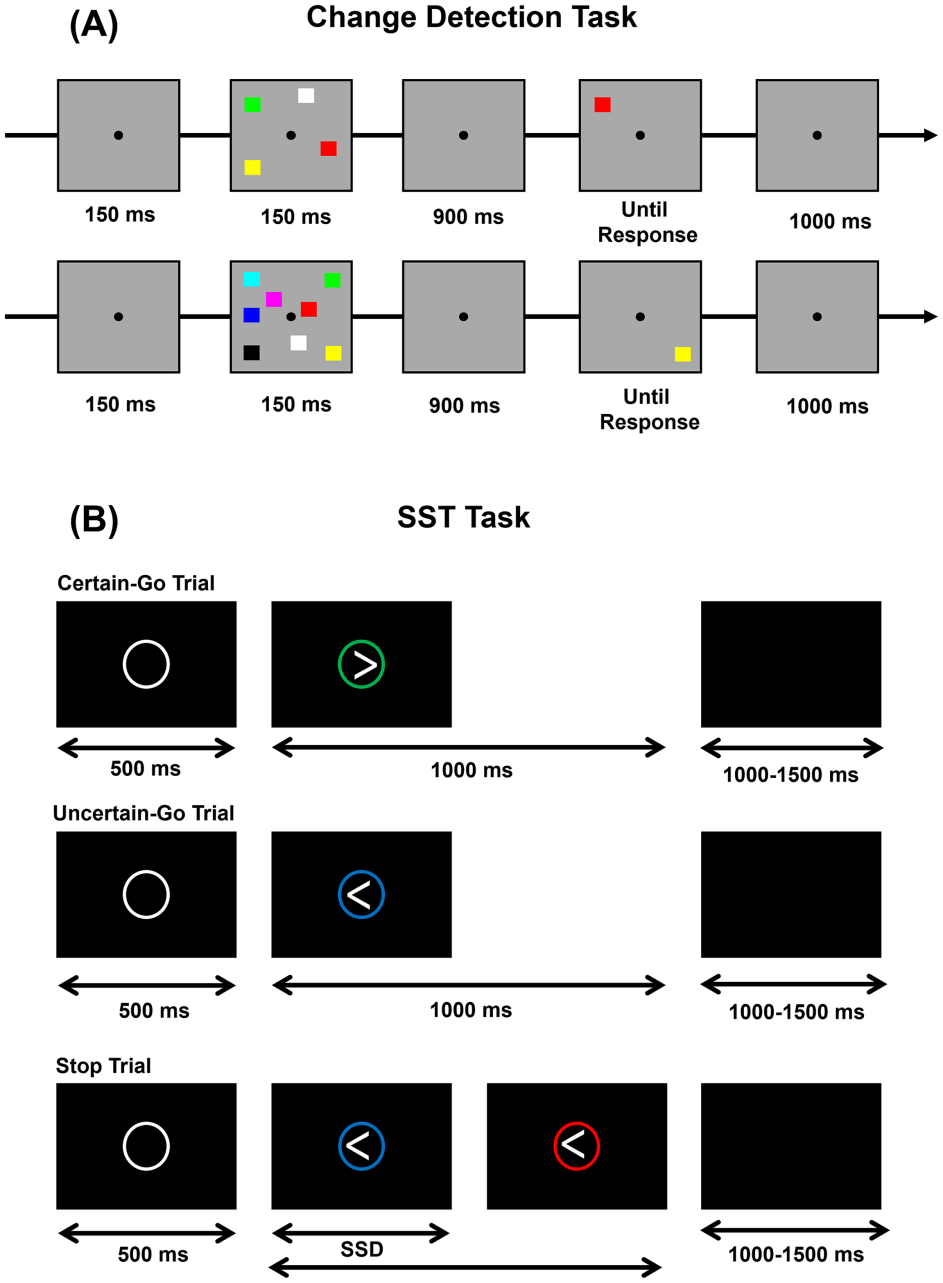
Experimental paradigms. **(A)** Change detection task. **(B)** Stop-signal task.

All experimental tasks were administered on a desktop computer equipped with a 60 Hz LCD monitor, with responses collected using a standard keyboard. Participants were seated approximately 60 cm from the screen in a quiet, dimly lit laboratory room to minimize external distractions. Visual WMC was assessed using CDT (Daniel et al., 2016; Balaban et al., 2019; Truong et al., 2022; Zhao et al., 2023) (Figure 1A). The visual stimuli were colored squares drawn from a set of nine distinct colors: red (RGB: 255, 0, 0), green (0, 255, 0), blue (0, 0, 255), yellow (255, 255, 0), magenta (255, 0, 255), cyan (0, 255, 255), orange (255, 128, 0), black (0, 0, 0), and white (255, 255, 255). Each trial presented either 4 or 8 squares randomly positioned within an invisible 4 × 4 grid centered on a gray background (RGB: 169, 169, 169), with a minimum center-to-center distance of 2.4° of visual angle to prevent overlap. Each trial began with a central fixation cross presented for 150 ms, followed by the memory array (150 ms). After a 900 ms delay period during which a black dot was displayed at the center, a probe square appeared at one of the previous locations. Participants indicated whether the probe color matched the corresponding memory array square by pressing the “F” key for a match or the “J” key for a non-match. The response period was self-paced. Each trial concluded with a 1,000 ms inter-trial interval displaying a blank gray screen. Visual WMC (*K*) was calculated for each set size using Cowan’s formula (Xu et al., 2018; Feuerstahler et al., 2019) (Equation 1):


K=Setsize×(Hitrate−False alarm rate) (1)

Participants’ performance was measured by the hit rate (the proportion of correct responses on match trials) and the false alarm rate (the proportion of incorrect responses on non-match trials). The experiment began with 20 practice trials, followed by two formal blocks of 60 trials each. Stimulus presentation and behavioral data collection [accuracy and reaction times (RTs)] were conducted using E-Prime 3.0 (Psychology Software Tools, Pittsburgh, PA).

#### 2.2.3 SST task

The SST employed in this study incorporated two conditions varying in stop-signal probability: a 0% stop-signal condition and a 25% stop-signal condition (Figure 1B). All procedures were conducted using the same apparatus as described above. In the 0% stop-signal condition, all trials were designated as Certain-Go trials. Each trial began with a 500 ms fixation (white circle), followed by a green circle containing a left- or right-pointing arrow presented for 1,000 ms. Participants were instructed to respond as quickly and accurately as possible according to the arrow direction. Trials ended with a blank screen displayed for a randomized duration between 1,000 and 1,500 ms. In the 25% stop-signal condition, each block consisted of 75% Uncertain-Go trials and 25% Stop trials. On Uncertain-Go trials, participants responded to a blue circle. On Stop trials, the blue circle changed to red after a stop-signal delay (SSD), signaling participants to inhibit their response. The initial SSD was set at 250 ms and dynamically adjusted using a staircase tracking procedure: SSD increased by 50 ms following successful inhibition and decreased by 50 ms following failed inhibition, converging on an approximate 50% inhibition rate. Stop trials were pseudo-randomly distributed, ensuring that the first trial of a block was never a stop trial and no two stop trials occurred consecutively.

Each participant completed six blocks, each consisting of 125 trials (25 Certain-Go, 75 Uncertain-Go, 25 Stop trials). Prior to the formal experiment, participants completed a practice session with 5 Certain-Go, 15 Uncertain-Go, and 5 Stop trials Stimulus presentation and behavioral data collection were using E-Prime 3.0 (Psychology Software Tools, Pittsburgh, PA).

### 2.3 Behavioral data analysis

All behavioral data were subjected to comprehensive statistical analyses. The primary variables included the ERQ subscale scores for cognitive reappraisal and expressive suppression, WMC (*K*) derived from the CDT, and the mean RTs for each condition of the SST. The normality of all continuous variables was examined using the Shapiro–Wilk test. All variables satisfied the normality assumption (all *p*s > 0.05), supporting the use of parametric statistical methods.

Paired-sample *t*-tests were conducted to compare mean reaction times across the different SST conditions. Pearson correlation coefficients were calculated to examine bivariate associations among study variables. Variables showing significant bivariate correlations were subsequently entered into multiple linear regression models to examine the unique predictive relationships among emotion regulation strategies, WMC, and cognitive control indices. The robustness and generalizability of these regression models were further examined using leave-one-out cross-validation (LOOCV), providing a stringent safeguard against Type I errors and ensuring the reliability of the predictive findings.

All statistical analyses were conducted using SPSS version 26.0 (IBM Corp., Armonk, NY, United States), except for regression modeling and validation analyses, which were implemented in MATLAB R2023a (MathWorks Inc., Natick, MA, United States).

## 3 Results

Descriptive statistics for the behavioral and cognitive measures are presented in Table 1. For emotion regulation strategies, cognitive reappraisal was reported at a significantly higher level (*M* = 4.76, SEM = 0.10) than expressive suppression (*M* = 3.55, SEM = 0.14; *t* (65) = 9.43, *p* < 0.001). In the CDT, RTs were significantly longer under the high-load condition (*M* = 1001.94 ms, SEM = 32.98) compared to the low-load condition (*M* = 893.38 ms, SEM = 27.22), *t* (65) = 8.45, *p* < 0.001, reflecting the robust load effect. The estimated mean WMC was *K* = 2.68 (SEM = 0.10). In SST, participants exhibited significantly longer RTs on Uncertain-Go trials (*M* = 558.19 ms, SEM = 6.38) compared to Certain-Go trials (*M* = 408.53 ms, SEM = 6.47), *t* (65) = 22.08, *p* < 0.001. The RT difference between Uncertain-Go and Certain-Go conditions (149.66 ± 6.78 ms) was operationalized as the PC effect.

**Table 1:** Descriptive statistics of behavioral and cognitive measures.

Measures	MIN	MAX	AVG	SEM
Emotion regulation strategies (1–7 scale)				
Expressive suppression	1.000	6.500	3.553	0.142
Cognitive reappraisal	2.833	6.500	4.763	0.101
Change detection task				
Load4 RT (ms)	586.319	1618.622	893.382	27.218
Load8 RT (ms)	596.333	1621.056	1001.938	32.984
Working memory capacity (K)	1.200	4.533	2.684	0.102
SST task (ms)				
Certain-Go RT	308.586	548.022	408.530	6.468
Uncertain-Go RT	438.573	707.595	558.186	6.376
SSRT	202.960	471.680	327.831	7.736
PC effect	11.666	261.963	149.656	6.777

**Figure 2:**
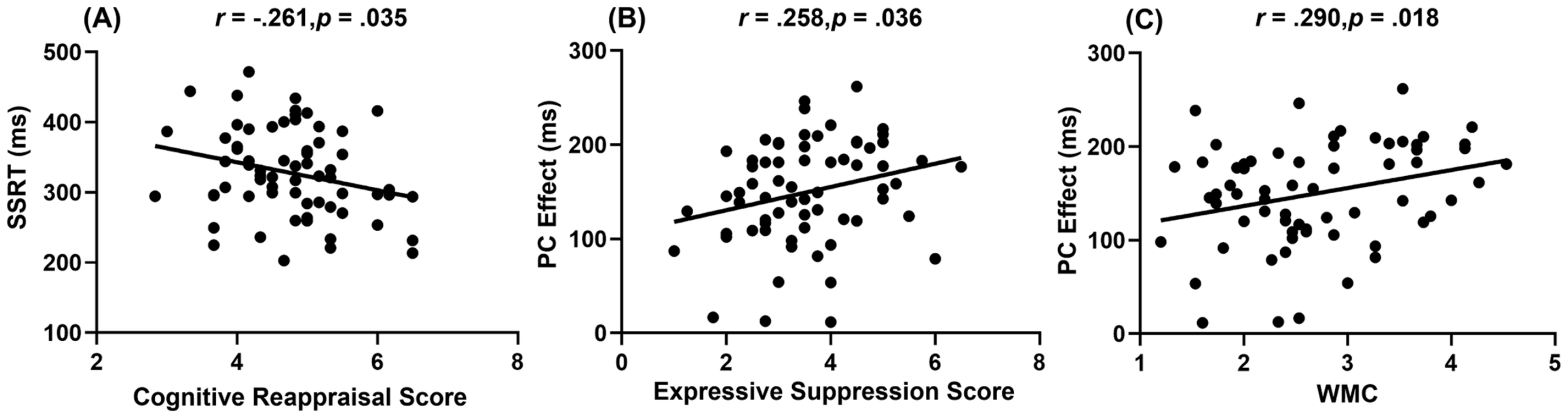
Correlations among WMC, emotion regulation strategies, and inhibitory control. **(A)** A significant negative correlation was observed between cognitive reappraisal scores and SSRT. **(B)** A significant association between expressive suppression and the PC effect. **(C)** A significant association between WMC and the PC effect. The black-filled circles denote the participants.

All variables satisfied normality assumptions (all *p*s > 0.05), and variance inflation factors (VIFs) below 1.4 indicated no multicollinearity. The correlations among working memory capacity (WMC), emotion regulation strategies, and inhibitory control are presented in Table 2. Significant correlations were observed between several cognitive and behavioral measures. WMC was positively correlated with the proactive control (PC) effect (*r* = 0.29, *p* = 0.018), indicating that higher WMC was associated with greater proactive control. Cognitive reappraisal scores were negatively correlated with SSRT (*r* = −0.26, *p* = 0.035), suggesting more efficient reactive control among individuals who reported greater use of cognitive reappraisal. Expressive suppression was positively correlated with the PC effect (*r* = 0.26, *p* = 0.036). In addition, the PC effect was positively correlated with SSRT (*r* = 0.34, *p* = 0.006), indicating that individuals showing greater proactive control benefits also tended to exhibit longer reactive inhibition times. These significant correlations are presented in Figure 2. Cognitive reappraisal and expressive suppression were moderately positively correlated (*r* = 0.48, *p* < 0.001). In contrast, WMC was not significantly correlated with SSRT (*r* = −0.10, *p* = 0.428). Cognitive reappraisal was not significantly associated with the PC effect (*r* = 0.16, *p* = 0.212), and expressive suppression showed no significant relationship with SSRT (*r* = 0.10, *p* = 0.412).

**Table 2:** Correlations among WMC, emotion regulation strategies, and inhibitory control.

Variable	WMC	CR	ES	PC effect	SSRT
WMC	—				
CR	0.07	—			
ES	0.11	0.48***	—		
PC effect	0.29*	0.07	0.26*	—	
SSRT	−0.05	−0.26*	0.10	0.34**	—

*N* = 66; WMC, working memory capacity; CR, cognitive reappraisal; ES, expressive suppression; PC effect, proactive control; SSRT, stop signal reaction time; *p < 0.05; **p < 0.01; ***p < 0.001.

**Figure 3:**
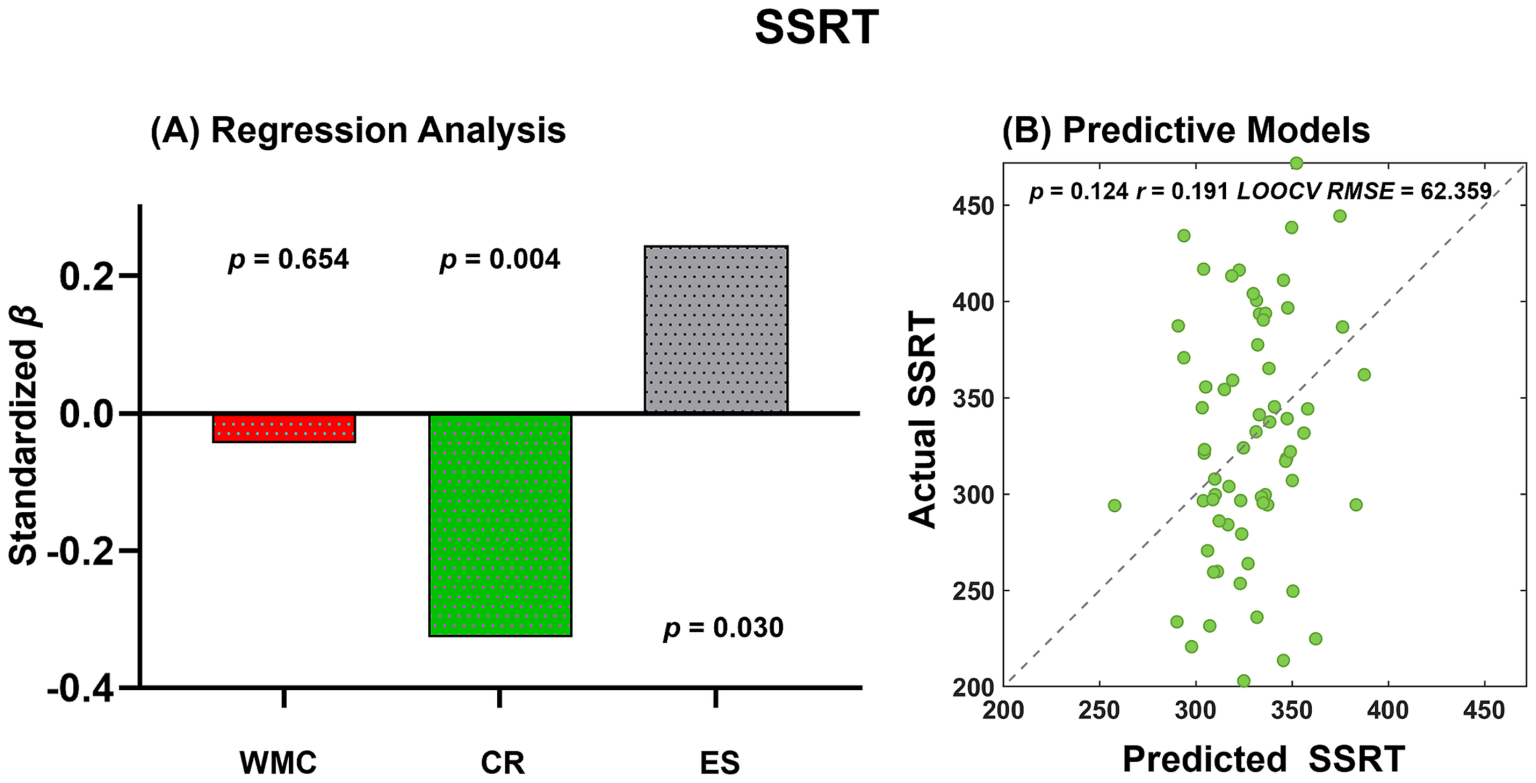
Regression analyses predicting SSRT. **(A)** Standardized regression coefficients for WMC, cognitive reappraisal, and expressive suppression. **(B)** The relationship between the predicted and actual SSRT. RMSE, mean square error of predicting the model. The green-filled circles denote the participants.

Multiple linear regression analyses were conducted to examine the unique predictive roles of WMC and emotion regulation strategies in cognitive control. Two separate models were tested, one predicting the PC effect and one predicting SSRT. Leave-one-out cross-validation (LOOCV) was employed to assess model generalizability. The regression model predicting SSRT was statistical significance, *F* (3, 62) = 3.30, *p* = 0.026, *R*^2^ = 0.14 (Figure 3). Cognitive reappraisal was a significant negative predictor (*β* = −0.33, *p* = 0.004), indicating that greater use of this emotion regulation strategy was associated with more efficient reactive control. Expressive suppression was a significant positive predictor (*β* = 0.24, *p* = 0.030), suggesting that higher use of this strategy was linked to less efficient reactive control. WMC did not significantly predict SSRT (*β* = −0.04, *p* = 0.654). However, LOOCV analysis indicated that the model’s predictive validity was limited, with the correlation between predicted and observed SSRT was not statistical significance (*r* = 0.19, *p* = 0.124, RMSE = 62.4 ms), suggesting that the model’s ability to generalize to new samples may be constrained.

**Figure 4:**
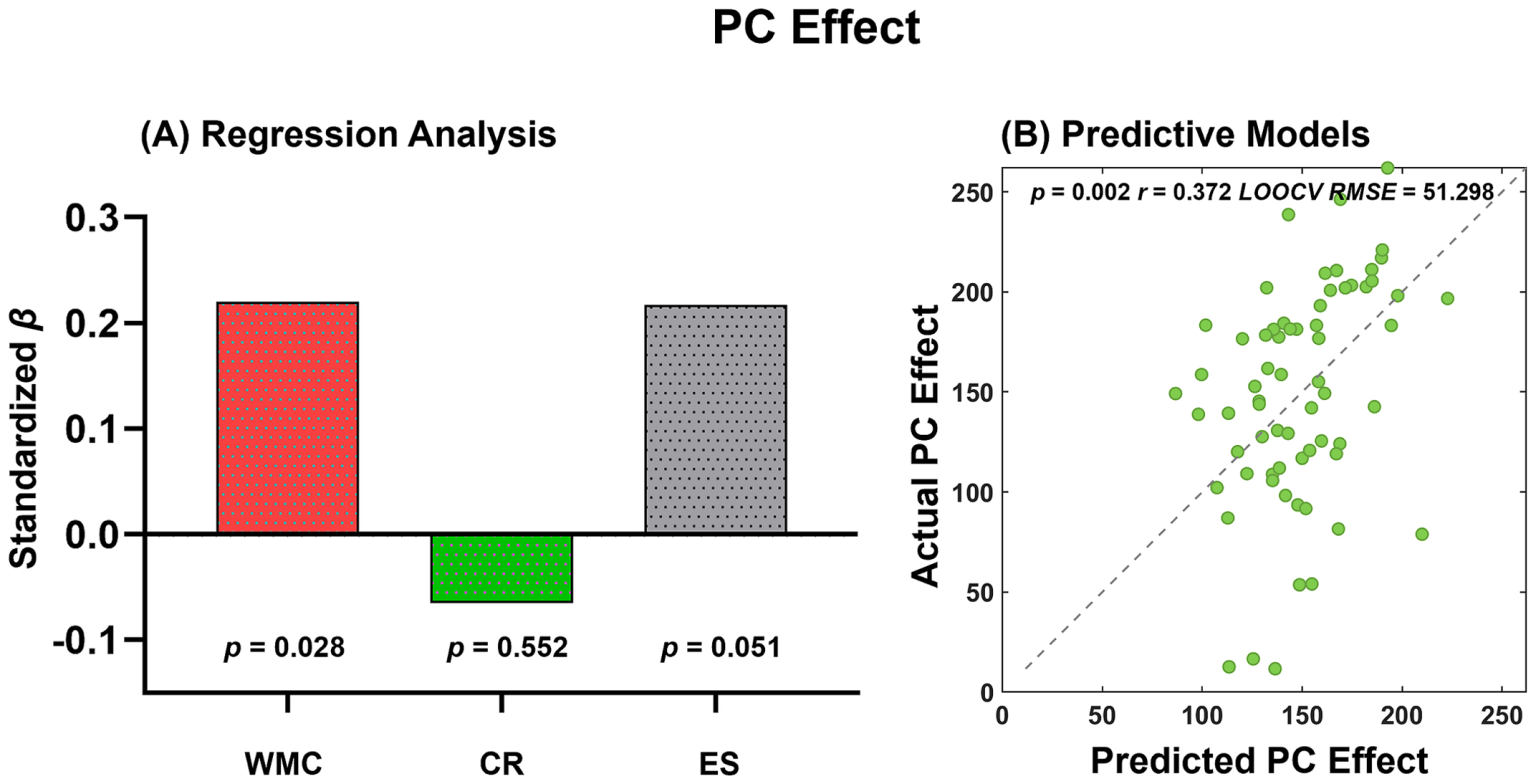
Regression analyses predicting the PC effect. **(A)** Standardized regression coefficients for working memory capacity (WMC), cognitive reappraisal, and expressive suppression. **(B)** The relationship between the predicted and actual PC effect. The green-filled circles denote the participants.

The regression model predicting the PC effect was statistically significant, *F* (3, 62) = 3.40, *p* = 0.023, *R*^2^ = 0.14 (Figure 4). WMC emerged as a significant positive predictor (*β* = 0.22, *p* = 0.028), indicating that individuals with higher WMC demonstrated stronger proactive control. Expressive suppression showed a marginally significant positive effect (*β* = 0.22, *p* = 0.051), while cognitive reappraisal did not significantly predict the PC effect (*β* = −0.07, *p* = 0.552). LOOCV analysis confirmed the model’s predictive validity, with a significant correlation between predicted and observed PC effect values (*r* = 0.37, *p* = 0.002, RMSE = 51.3 ms). The predicted versus actual values plot demonstrated reasonable model fit, with most data points clustering near the identity line.

## 4 Discussion

This study examined how WM and emotion regulation strategies jointly contribute to proactive and reactive cognitive control within the DMC framework. By employing a modified SST alongside a CDT and the ERQ, we were able to disentangle the contributions of proactive and reactive control and quantify individual differences in their efficiency. The results revealed differential patterns of prediction. Specifically, cognitive reappraisal significantly accounted for variance in SSRT, whereas WM significantly linked to the PC effect. Together, these findings provide behavioral evidence that cognitive resources and affective regulation differentially relate to proactive and reactive cognitive control, supporting the DMC framework’s assumption of flexible, interactive mechanisms of cognitive control (Braver, 2012; Luo et al., 2015; Braver et al., 2021; Badre, 2025).

Reactive control was significantly related to cognitive reappraisal. Participants who habitually employed cognitive reappraisal demonstrated shorter SSRTs, indicating more efficient inhibitory control. These results align with previous research indicating that cognitive reappraisal is associated with adaptive goal-directed behavior and effective self-regulation (Butler et al., 2003; Ochsner and Gross, 2005; Verbruggen and Logan, 2008b; Li et al., 2021a). One possible explanation is that emotion regulation and reactive control may rely on partially overlapping neural mechanisms. Neuroimaging studies highlight the critical role of the right inferior frontal gyrus (rIFG) in reactive control and stopping behavior (Niendam et al., 2012; Aron et al., 2014), as well as its essential function in emotion regulation (Li et al., 2021a), which may provide a potential neural basis for the observed association. As an antecedent-focused strategy, cognitive reappraisal is thought to operate early in processing and is generally associated with effective emotional and cognitive functioning. In contrast, expressive suppression, another trait-level emotion regulation strategy, did not show a significant zero-order correlation with SSRT. Its positive regression coefficient suggests that, after accounting for overlapping variance with working memory capacity and cognitive reappraisal, expressive suppression may capture unique aspects of self-regulatory tendencies that are relevant to reactive control. As a response-focused strategy, expressive suppression requires sustained inhibition of emotional expression throughout the emotional episode (Gross and John, 2003; Gross, 2015), imposing continuous regulatory effort that can interfere with the rapid engagement of inhibitory mechanisms necessary for efficient reactive control (Jaffard et al., 2008; Luo et al., 2015). It should also be noted that SSRT is not a process-pure measure of inhibitory control. Performance on the stop-signal task may additionally reflect attentional monitoring, motor response preparation, and speed-accuracy trade-offs (Logan and Cowan, 1984; Verbruggen and Logan, 2008a,b; Badre, 2025). Therefore, the observed association between emotion regulation strategies and SSRT may partly reflect shared variance with these attentional and motor components of reactive control.

Proactive control was significantly linked to WM. The PC effect is thought to involve the maintenance and updating of task-relevant information, which constitute central functions of WM (Richards and Gross, 2000; Engle, 2002; McCabe et al., 2010; Cowan, 2016; Rosales et al., 2022). Individuals with higher WMC are better able to sustain goal-relevant representations during cue-target intervals, facilitating more effective preparation for upcoming task demands (Richmond et al., 2015; Arslan et al., 2024; Lin et al., 2024). This cognitive advantage manifests behaviorally as a more pronounced PC effect, reflecting enhanced proactive engagement (Braver et al., 2007; Gilbert et al., 2010; Irlbacher et al., 2014; Rosales et al., 2022; Rónai et al., 2024). Neuroimaging studies further suggest that this sustained goal maintenance is supported by the dorsolateral prefrontal cortex (DLPFC), which plays a critical role in maintaining goal representations and biasing attention toward task-relevant information (Miller and Cohen, 2001; Curtis and D’esposito, 2003; Li et al., 2021b; Xu et al., 2023). Thus, higher WM may be linked to greater availability of cognitive resources that support the continuous activation and maintenance of task goals during proactive control. Importantly, these findings are supported by the study’s statistical power. Given the current sample size and model design, the analyses are sufficiently powered to detect medium-to-large effects, indicating that the observed relationships are reliable.

Expressive suppression showed a marginally significant positive correlation with the PC effect in the regression model, although this effect did not reach conventional statistical significance. This pattern, while preliminary, is consistent with theoretical perspectives suggesting that habitual use of expressive suppression may be associated with greater inhibitory control capacities that support proactive control (Richards and Gross, 2000; Ochsner and Gross, 2005; Rónai et al., 2024). Expressive suppression requires sustained inhibition of emotional expression throughout an emotional episode (Gross, 1998; Gross and John, 2003; Kragel et al., 2018), a process that imposes substantial cognitive costs and engages executive control processes similar to those required for proactive control (Richards and Gross, 2000; Ochsner and Gross, 2005; Nigg, 2016). Consequently, individuals who habitually employ expressive suppression may develop enhanced capacity for sustained cognitive engagement. However, this finding remains tentative given the modest sample size and marginal significance.

Moreover, the significant positive correlation between the PC effect and SSRT suggests that proactive and reactive control processes may be functionally related rather than entirely independent mechanisms. This finding provides empirical support for the DMC framework (Braver, 2012), which conceptualizes cognitive control as a dynamic system allowing individuals to flexibly shift between sustained (proactive) and transient (reactive) modes depending on situational demands. Consistent with recent evidence (Luo et al., 2015; Chiew and Braver, 2017; Viviani et al., 2024; Rincón-Pérez et al., 2025), the distinct patterns observed in how WM and emotion regulation strategies predict different control modes may reflect individual differences in control deployment preferences (Luo et al., 2015; Lin et al., 2024). Notably, the current findings are derived from a sample of healthy young university students. This specific population typically exhibits an optimal cognitive profile characterized by peak processing speed and superior attentional control compared to middle-aged and older adults (Wang et al., 2024; Waring and Hartling, 2025). Given that aging is often associated with a decline in proactive control resources and a potential shift toward reactive strategies, the predictive power observed here may represent an upper bound of cognitive efficiency.

Collectively, the current findings potentially align with the neurobiological distinction between proactive goal maintenance and reactive response suppression within the DMC framework (Braver, 2012; Niendam et al., 2012). This dissociation suggests that working memory and emotion regulation strategies may differentially modulate these separable control modes through their respective associations with DLPFC and rIFG-mediated processes. Additionally, these findings contribute to understanding cognitive control deficits in clinical populations with impairments in WM and emotion regulation, such as ADHD, anxiety, and depression (Kragel et al., 2018; Curtin et al., 2019; Gao et al., 2024). Specifically, targeting these distinct pathways by using working memory training for proactive deficits and emotion regulation strategies for reactive impulsivity could provide a more tailored approach to clinical intervention.

Several limitations should be considered when interpreting these findings. First, although this study treated emotion regulation as a predictor of cognitive control, the possibility of reverse causation cannot be excluded. Individual differences in baseline executive function may influence the choice of regulatory strategies. For example, greater inhibitory control or working memory capacity may provide the cognitive resources necessary for effective reappraisal, whereas limitations in executive function may predispose individuals to rely on response-focused suppression (Toh and Yang, 2024). Future research could consider conducting a reverse-predictive experiment by first measuring baseline proactive and reactive control capacities, and then designing working memory and emotion regulation tasks to assess the use of emotion regulation strategies and working memory performance, in order to further explore the relationships among the three. Second, while the sample size (*N* = 66) is sufficient for detecting main correlations, it may affect the strength of the predictive models. Larger sample sizes in future studies are necessary to confirm the robustness of these predictive relationships. Third, as previously mentioned, the specific participant group in this study may restrict the generalizability of the findings (Luo et al., 2015; Zhang and Wang, 2022). Future research should consider a more diverse sample to enhance the generalizability of the results. Fourth, the LOOCV results for the SSRT model were not significant, suggesting that the model lacks strong predictive validity. Future research should validate the relationship between emotion regulation strategies and cognitive control in independent samples to ensure the generalizability of these findings. Finally, the ERQ assesses the tendency to use cognitive reappraisal and expressive suppression, but does not measure the emotion regulation ability or effectiveness (Gross, 2015). Future research should incorporate performance-based behavioral or physiological measures to determine whether these findings are driven by strategy preference or the ability to regulate emotions effectively.

## 5 Conclusion

In conclusion, this study examined the distinct contributions of WMC and habitual emotion regulation strategies to proactive and reactive cognitive control within the DMC framework. The results revealed differential predictive patterns, with WMC was associated with proactive control and cognitive reappraisal was associated with more efficient reactive control. While expressive suppression showed a marginal contribution to proactive control, it emerged as a unique predictor of less efficient reactive control only within the multivariate regression model, indicating a suppressor effect. The positive correlation between the PC effect and SSRT indicates partial interdependence between proactive and reactive control. Overall, these findings provide behavioral evidence that cognitive resources and emotion regulation strategies show distinct yet complementary links to cognitive control, highlighting the dynamic interplay between cognitive and affective systems in goal-directed behavior.

## Data Availability

The data that support the findings of this study are available on request from the corresponding authors. Requests to access the datasets should be directed to Ping Xu, xp@cmc.edu.cn.
